# Animal choruses emerge from receiver psychology

**DOI:** 10.1038/srep34369

**Published:** 2016-09-27

**Authors:** Michael D. Greenfield, Yareli Esquer-Garrigos, Réjane Streiff, Virginie Party

**Affiliations:** 1 Institut de recherche sur la biologie de l’insecte (IRBI), CNRS UMR 7261, Parc de Grandmont, Université François Rabelais de Tours, 37200 Tours, France; 2 INRA, UMR 1062 CBGP, Campus International de Baillarguet, F-34988 Montferrier-sur-Lez, France; 3 INRA, UMR 1333 DGIMI, Université de Montpellier, Place Eugène Bataillon, 34095 Montpellier cedex 5, France

## Abstract

Synchrony and alternation in large animal choruses are often viewed as adaptations by which cooperating males increase their attractiveness to females or evade predators. Alternatively, these seemingly composed productions may simply emerge by default from the receiver psychology of mate choice. This second, emergent property hypothesis has been inferred from findings that females in various acoustic species ignore male calls that follow a neighbor’s by a brief interval, that males often adjust the timing of their call rhythm and reduce the incidence of ineffective, following calls, and from simulations modeling the collective outcome of male adjustments. However, the purported connection between male song timing and female preference has never been tested experimentally, and the emergent property hypothesis has remained speculative. Studying a distinctive katydid species genetically structured as isolated populations, we conducted a comparative phylogenetic analysis of the correlation between male call timing and female preference. We report that across 17 sampled populations male adjustments match the interval over which females prefer leading calls; moreover, this correlation holds after correction for phylogenetic signal. Our study is the first demonstration that male adjustments coevolved with female preferences and thereby confirms the critical link in the emergent property model of chorus evolution.

Choruses of acoustic species are among the more spectacular displays of animal behavior in the natural world^1^. Beyond the sheer number of participating individuals and the sound intensity of their collective broadcast, animal choruses may also feature striking harmonization between individual singers^2^. Such precision is especially evident in species where individuals maintain a particular call rhythm when singing in solo and then adjust the phase and/or rate of their rhythm when accompanied by neighbors. The overall result of these individual adjustments may be in-phase synchrony, out-of-phase alternation, or an elaborate combination of both^3^. Analogous cases of synchrony are also reported in species using visual signals^4^,^5^,^6^.

Although the neuroethological mechanisms by which individuals may adjust their song are rather well-known^3^,^7^,^8^, understanding why animals generate choruses that feature synchrony and alternation has evaded rigorous testing and is largely conjectural. Most of the conjectures propose that synchrony and alternation are specialized adaptations through which cooperating males directly or indirectly increase their mating success or reduce their vulnerability to natural enemies attracted to the songs^9^,^10^. For examples, both types of chorusing patterns may reduce signal interference and thereby offer females improved perception of call properties, and they may also increase the combined sound intensity perceived from a group of singers^11^,^12^. Additionally, synchrony may prevent predators from localizing any one singer within a dense chorus. Support for the reduced signal interference^12^,^13^,^14^ and increased group intensity^12^,^15^ hypotheses has been found in a few species, but in general the purported benefits of the displays are difficult to examine and have not been checked.

An alternative to the adaptationist paradigm above, and a key to its thorough examination, is the null hypothesis that the collective singing patterns in choruses, so conspicuous to human observers, simply emerge from the ‘receiver psychology’^16^ of female perception and preference^10^,^15^,^17^. For want of explicit experiments conducted on appropriate chorusing species, the null hypothesis has been especially conjectural. But recent neuroethological studies of female preference point toward a specific 3-step pathway along which basic perception and sexual selection could ultimately lead to chorusing: In various acoustic species females prefer male calls that precede a neighbor’s call by a brief interval^18^, a variant of the precedence effects known from psychoacoustic research^19^. The next step in the pathway is the finding in many species which sing rhythmically that when a male hears a song stimulus, he delays his subsequent call via a mechanism involving momentary inhibition of his central rhythm generator and resetting of his phase relative to that stimulus^9^,^10^ (Fig. 1). Finally, when multiple males use equivalent mechanisms an expansive chorus comprised of synchrony and/or alternation may arise^20^ (Supplementary note S1). These collective outcomes are predicted by Monte Carlo simulation, and they are consistent with observations of synchrony and alternation in various acoustic insects and anurans^21^ (Fig. 2). Importantly, the display can be generated in the absence of any selection expressly favoring synchrony or alternation.

The 3-step pathway along which chorusing may emerge assumes that male resetting is a competitive mechanism favored by the selection imposed by female preference for leading calls. Simulations showing that males who inhibit and reset their call rhythms broadcast more leading calls and fewer following ones, and attract more females, than males with comparable song but who do not reset^21^ are in agreement with this assumption. Nonetheless, the inferred coevolution between the sensorimotor activity of male resetting and the female preference for relative call timing has not been established empirically in any acoustic species. Owing to this uncertainty, reflecting the lack of experiments probing the critical relationship between male and female timing parameters, the emergence of chorusing has remained speculative.

To test the null, or emergent property, hypothesis we chose to study *Ephippiger diurnus*, a European katydid that offered a special opportunity to analyze the apparent relationship between male resetting adjustments and female preference. *E. diurnus* are distributed in genetically distinct, geographically isolated populations^22^ to which comparative phylogenetic methods could be applied. The various populations exhibit divergent male songs, chorusing patterns (Fig. 2), and female preferences for male song^23^,^24^. Male *E. diurnus* effect rhythm adjustments by inhibitory-resetting in response to the playback of song stimuli or to neighbors^21^, and females have moderate to strong preferences for leading calls^25^. Thus, we could determine whether male resetting and female preference have actually coevolved or only represent a spurious association.

We reasoned that if male resetting adjustment evolved in response to female preference for leading calls, the timing of these 2 traits would be interconnected such that males seldom produce calls that most local females perceive as following ones and therefore ignore. As in other acoustic species using resetting mechanisms, male *E. diurnus* initiate few or no calls during a delay interval of minimum length *m* following the onset of a neighbor’s call or a synthetic song stimulus^21^ (Fig. 3A,B). Females show a pronounced preference for a leading over a following call if the latter begins during an interval *f* after the onset of the leading call (Fig. 3C). When *m* is ≥ *f*, males will avoid broadcasting ineffective following calls, and when *m* = *f*, males will maximize their call rate and improve their chance of broadcasting leading calls as well. We sampled 17 *E. diurnus* populations from southern and central France and northeastern Spain chosen to cover a broad geographic range, genetic variation as suggested by earlier phylogeographic studies^22^, and a diversity of male songs and chorusing patterns (Fig. 4A). In each population we determined *m* by testing male acoustic responses to the playback of song stimuli and *f* by testing female movement toward the leading of 2 song stimuli. We also developed a neighbor-joining (NJ) tree, based on microsatellite markers^26^, to serve as our ‘working phylogeny’ of *E. diurnus* populations.

## Results

We report that the minimum delay *m* in male calling following a song stimulus is strongly correlated with, and roughly matches, the maximum separation *f* between 2 calls for which females prefer the leading one (ρ = 0.87, p < 0.001; Fig. 4B). *m* was slightly shorter than *f* in 13 of 17 populations, possibly reflecting a conflict that males face between producing calls deemed leading by at least some females and avoiding the production of following ones and that it is resolved in favor of the former.

In further analyses we dealt with several factors that potentially generated the matching of male and female timing parameters as an artifact. First, we applied the method of independent contrasts (PIC) to our NJ tree of populations to remove any phylogenetic signal from the *m*-*f* correlation^27^. Under most circumstances inter-population gene flow would invalidate the use of PIC to treat intra-specific relationships^28^,^29^, but the negligible migration in *E. diurnus*, which are flightless, and the distinctive population genetic structure fully justify its application here. Our NJ tree (Fig. 5A) clearly reveals a branching topology that is consistent, in its details, with another tree based on COI^30^, indicating that our working phylogeny is most probably an accurate depiction of the true one. To be conservative, we also analyzed our microsatellite data with a Bayesian clustering protocol to identify genetically distinct population clusters and assign individuals to these entities (Fig. 6). We then created a NJ tree for the 7 strongly differentiated clusters identified (Fig. 5C) and again applied the PIC correction. We found that the *m*-*f* correlation remained highly significant following PIC correction whether applied to the tree of all 17 populations sampled or to the revised tree of 7 genetically distinct clusters (Fig. 5B,D). Thus, the observed matching of male and female timing parameters is not a phylogenetic artifact of our sampled populations.

Second, we accounted for the possibility that both the male and female timing parameters simply reflect the call length (=syllable number) and free-running call rhythm in a population. In fact, the male timing parameter, *m*, is closely correlated with call length (ρ = 0.94; p < 0.001), a relationship that could allow the several outlier populations with long calls, and slow call rhythms, to unduly influence the *m*-*f* correlation. We therefore reanalyzed our data using only populations with relatively short calls, those with a mean of 1.0–2.1 syllables. In this reduced data set of 10 populations the *m*-*f* regression and correlation held (t = 2.64, p = 0.03; ρ = 0.69, p = 0.029; Fig. 4C), whereas the correlation between *m* and mean syllable number did not (t = 1.65, p = 0.14; ρ = 0.50, p = 0.14). Our analysis is the first confirmation that male rhythm adjustments have coevolved with female preferences for call timing.

## Discussion

**Figure 2:**
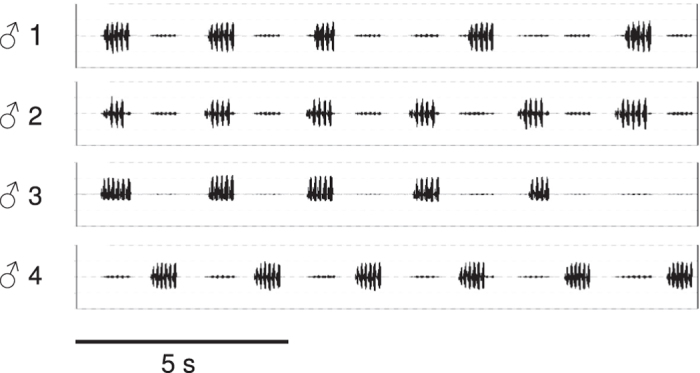
Elaborate chorusing in *E. diurnus*. Multi-channel oscillogram of a representative 15-s sample of calling recorded from a 4-male chorus in the Peyriac de Mer population (Fig. 4A). See Supplementary note S1 on the occurrence of both synchrony and alternation.

**Figure 4:**
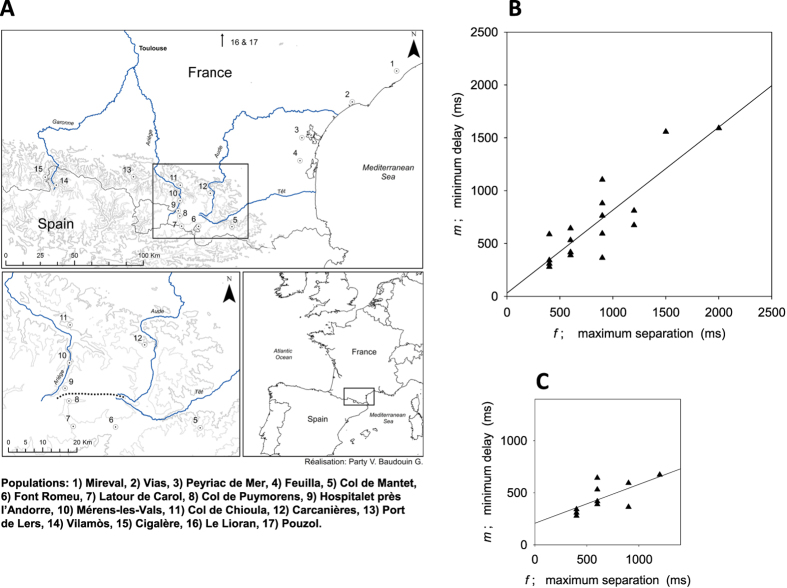
Correlation of male and female timing parameters across populations. **(A)** Map of southern France and northeastern Spain showing locations of the 17 *E. diurnus* populations sampled between 2012–2015 (see ref. 30); map was generated with ArcGIS version 10.0 for desktop, ESRI (http://www.esri.com/software/arcgis/arcgis-for-desktop). **(B)** Ordinary least-squares linear regression of *m* (minimum post-stimulus call delay in males; Fig. 3B) vs. *f* (maximum leader-follower call separation for which females prefer the leading call; Fig. 3C) for the 17 sampled populations (*m* = 31 + 0.79 *f*; t = 6.56, p < 0.001). **(C)** Ordinary least-squares linear regression of *m* vs. *f* as in Fig. 4b but restricted to those 10 populations where the mean call syllable number in males was ≤2.1 (*m* = 207 + 0.37 *f*). Stepwise linear regression (α to enter and α to remove = 0.15) of *m* on both *f* and mean syllable number yielded the above model that only included *f*.

**Figure 5:**
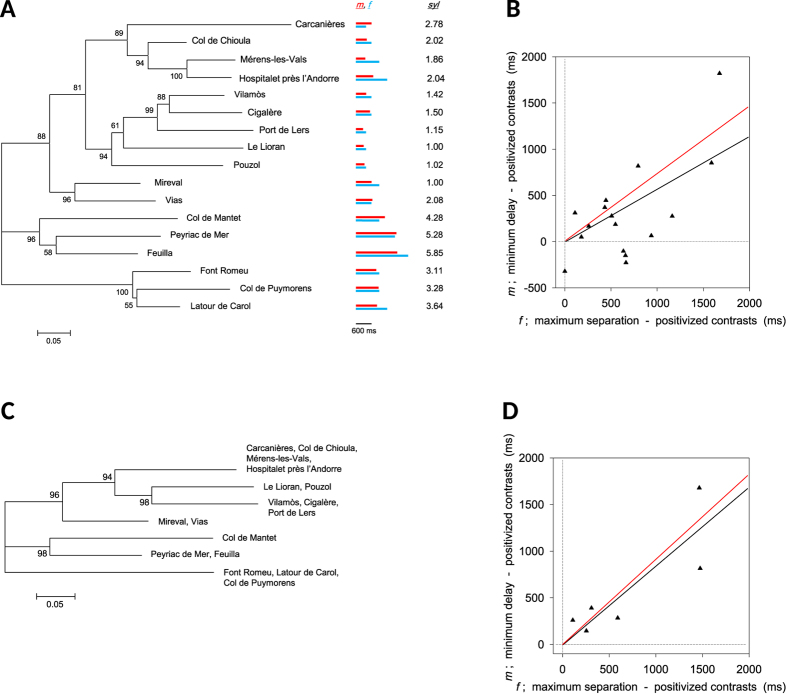
Comparative phylogenetic analysis of male and female timing parameters. **(A)** Unrooted neighbor-joining (NJ) tree of 17 *E. diurnus* populations from southern France and northeastern Spain (Fig. 4A) generated from microsatellite loci. Scale at bottom left indicates 0.05 nucleotide substitutions per site; values over branches represent posterior probabilities. Red and blue bars to the right of each population indicate the values of *m* (minimum post-stimulus call delay in males) and *f* (maximum leader-follower call separation for which females prefer the leader); see Figs 3B,C and 4B. *syl* is mean syllable number in the male call. **(B)** Correlation between *m* and *f* among the 17 populations corrected by phylogenetically independent contrasts (PIC). Black line is ordinary least-squares linear regression through the origin for the 16 standardized, positivized contrasts (ρ = 0.76, p < 0.001; *df* reduced by 2 to account for polytomy); red line is reduced major axis regression. **(C)** Unrooted NJ tree of 7 genetically distinct clusters determined via Bayesian clustering (Fig. 6). Each cluster comprises 1–4 of the 17 populations. **(D)** Correlation between mean *m* and *f* among the 7 clusters from Fig. 5C corrected by PIC (ρ = 0.94, p < 0.01; *n* = 6 contrasts).

2 parallel studies of acoustic communication in *E. diurnus* reinforce our conclusion that temporally-structured chorusing can emerge from receiver psychology. A test of female preference among chorusing males whose calling differed in several acoustic parameters showed that call timing, i.e. the number of leading calls, was the most influential character on male attractiveness^31^. Males are evidently under strong selection pressure to adjust their calling with a resetting mechanism. And in playback tests of synthetic chorus stimuli imitating the alternation or synchrony that occur naturally (Fig. 2) vs. modified chorus stimuli that do not occur, females expressed no preference for the natural stimuli^30^. This result, which is comparable to findings in an experimental study of synchronized visual signaling in fiddler crabs^32^, argues against the signal interference and combined intensity hypotheses^12^ for the evolution of chorusing in *E. diurnus*. Similarly, the simultaneous occurrence of both alternation and synchrony in *E. diurnus* choruses (Fig. 2) is not consistent with explanations invoking enhanced defense against predators and parasites. These hypotheses propose that synchronous signals might reduce the cognitive ability of natural enemies to localize a prey or host individual, or that males that synchronize signals can thereby listen for the approach of natural enemies during the silent intervals between successive signals^9^,^10^. However, a high incidence of alternated signals, as in *E. diurnus* choruses, would greatly reduce these potential advantages. Regularly alternated signals are also explained as a specific adaptation allowing males to broadcast signals and to assess rival neighbors in the absence of acoustic interference^9^. But again, the high incidence of synchronized signals in *E. diurnus* choruses would severely mitigate these effects.

Our findings on *E. diurnus* best support the null, or emergent property, hypothesis in which female preferences for relative call timing select for male rhythm adjustments which collectively yield patterns of synchrony and alternation. We do not claim that all or even most chorusing, in singing insects or in any other animal group, simply emerges from receiver psychology. Rather, our experimental findings show that this origin is possible and is a viable alternative to the adaptationist model. In a broader context, the emergence of chorusing from receiver psychology is an example of a self-organized system, a process that originates in local interaction within pairs and triads of individuals and lacks central control^33^. Self-organization is responsible for many complex systems in biology, e.g. coordinated activity of animal groups. But unlike most of the systems under consideration, the *E. diurnus* males who generate a chorus do not benefit from their collective production *per se*, and no evidence suggests that the females who listen to choruses pay any particular attention to their overall pattern in choosing a mate^10^,^17^. Emergent phenomena such as *E. diurnus* chorusing thus play an important role in evolutionary biology by reminding us that not all complex behavior we observe represents a specialized adaptation to presumed selection pressures.

## Methods

### Sampling and playback experiments

In each *E. diurnus* population sampled males and females were collected and then tested at approximately the same time. In 14 of the 17 populations test insects were collected as nymphs or young adults in the field; in 3 populations they were the laboratory-reared offspring of insects collected in the field during the previous year. *n* = 8–18 males in all but 1 population (Cigalère), where *n* = 3; *n* = 6–18 females in all but the Cigalère population, where *n* = 4.

We determined the timing of the resetting mechanism in *E. diurnus* males by playback experiments conducted in the laboratory at 22–24 °C. The synthetic call stimulus used for playback with a given population was constructed from a natural call syllable having average acoustic characteristics in that population; this representative syllable was then digitally repeated to produce a call with a syllable rate and syllable number equivalent to the population mean values. Using a sound level meter (model 1982; General Radio; Concord, Massachusetts, U.S.A.) calibrated with a microphone sensitive to high frequency and a computer (see refs 31 & 34 for method), we adjusted the amplitude of the stimulus to 80 dB SPL (peak reading; 0 dB = 20 μPa) at the location of the test male; this amplitude value represented males singing approximately 1 m distant in the field. Males collected in the field were obtained as nymphs or young adults; they were at least 15 d post adult molt and had not mated for a minimum of 10 d at the time of testing.

We determined the timing of preference for leading, as opposed to following, calls in *E. diurnus* females by playback experiments conducted under the same laboratory conditions. 2 synthetic call stimuli broadcast in close succession served as the basic stimulus. For each of 6–8 different call separation intervals ranging from 30–2000 ms, a female was tested individually in 4 successive trials on a y-maze at which loudspeakers broadcasting leading and following calls separated by the given interval were placed by the left and right arms; broadcasts of leading and following calls were switched between the 2 arms in successive trials. The maze was constructed of wood and consisted of a base and two cylindrical 70-cm arms separated by 90° and angled upward at 30°. Laboratory walls surrounding the apparatus were covered with acoustic insulation foam to reduce echoes from the loudspeaker broadcasts. Females were placed at the base of the y-maze and allowed 2 min to move toward the fork and continue onto one of the arms, considered as her choice. The index of preference for a given leader-follower call separation in a population was determined as [(*p*_*L*_ − 0.5)/0.5], where *p*_*L*_ is the proportion of choices for the leader summed across the 4 trials and all test females. Thus, the index reflected both the proportion of females that preferred a leading call and the strength of each individual female’s preference. Because each sampled female was tested in the same number of trials, each individual female contributed equally to the population’s index. Call stimuli were the same as those used to test timing of the resetting mechanism in males. The call stimulus for a given population was copied to the second channel of a stereo sound file, and the relative timing of the 2 channels was adjusted to the desired call separation; this separation interval was measured from onset of the leader to onset of the follower. The repetition rate for the stereo call stimulus was set equivalent to the mean call rate observed among interacting males in the population. Females collected in the field were obtained as nymphs or young adults; they were at least 15 d post adult molt and had neither mated nor been exposed to males or male song for a minimum of 10 d at the time of testing. See ref. 30 for additional details on protocol.

### Molecular genetics and phylogeny

Genomic DNA was extracted from hind femora of 335 *E. diurnus* adults using the DNA Easy Blood and Tissue kit (Qiagen, France) following the manufacturer’s instructions. DNA quality and molecular weight were confirmed in 1% agarose gel. A novel set of polymorphic microsatellite markers was developed for *E. diurnus* through pyrosequencing technology (Genoscreen, Lille France)^26^. In total, 18 microsatellite loci, including 4 loci previously reported^35^,^36^, were amplified in 7–26 specimens from 17 localities using a cost-effective M13 fluorescent protocol^37^ and multiplexed PCR. Fragment analysis was conducted on a 3730 xl DNA analyzer (Applied Bio Systems; AB) using the GeneScan 500 LIZ (AB) as an internal size standard and 1 to 2 μl of PCR product (1:20 dilution). Genotypes were resolved in GeneMapper version 5.0 (AB). Allele frequencies were estimated in POPTREEW^38^, and an unrooted neighbor-joining (NJ) tree of populations was constructed based on the *D*_*A*_ distance; this distance has been found to be an appropriate measure for obtaining the correct topology of population trees based on microsatellite markers. The robustness of nodes was estimated using 10,000 bootstrap replicates.

Phylogenetic independent contrasts (PIC) were conducted in MESQUITE version 3.04^39^,^40^. The degrees of freedom used in evaluating correlation coefficients were adjusted for polytomy^41^.

**Figure 6:**
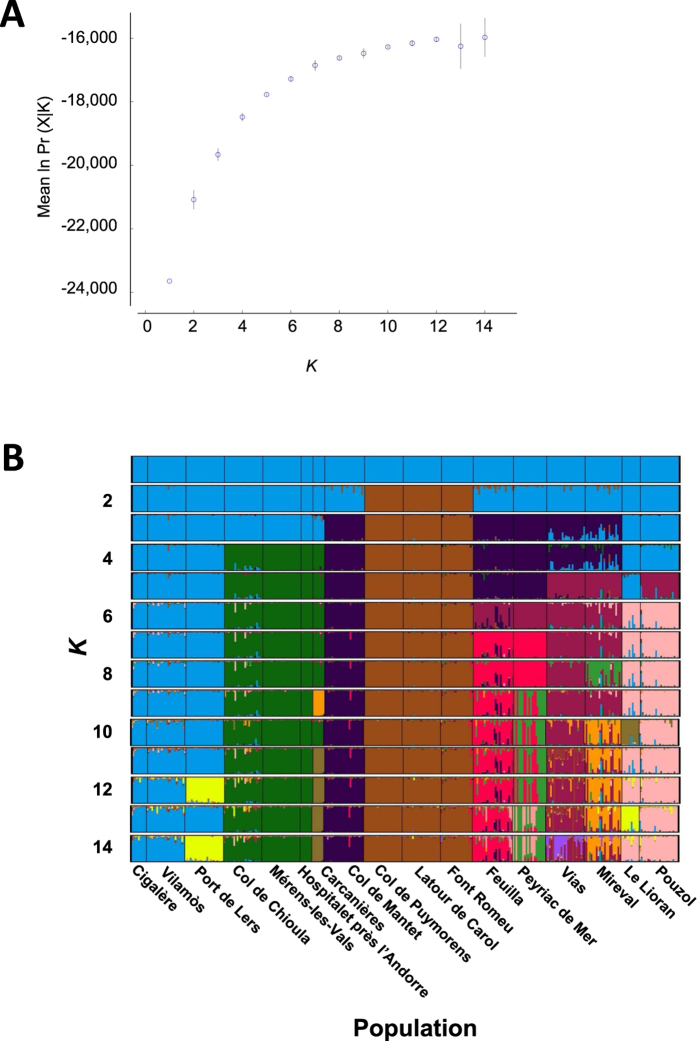
Clustering of genetically similar *E. diurnus* populations. **(A)** Mean and standard deviation of ln (probability of data) for each inferred number of clusters (*K* = 1–14) as estimated from Bayesian STRUCTURE analysis. Values are computed over 20 runs per *K*. **(B)** Bayesian STRUCTURE plots representing distinct clusters inferred from the 17 *E. diurnus* populations sampled (Fig. 4A). Each of the 14 horizontal panels represents a STRUCTURE plot based on a given number of inferred clusters (*K* = 1–14); in each plot distinct clusters are coded by a different color. The 335 individuals sampled from all 17 populations are each represented by a narrow vertical line, and the probability (*q* = 0 to 1) of an individual’s assignment to a distinct cluster is indicated by the height of the line segment bearing the color of that distinct cluster. Thus, individuals represented by lines composed of 2 or more segments of different colors may belong to the several distinct clusters whereas those represented by lines composed of a single color are likely to be pure. Fig. 5C depicts the distinct clusters inferred with *K* = 7.

We used a Bayesian clustering approach (STRUCTURE version 2.3.4^42^) to identify the most likely number of distinct clusters (*K*) in our dataset and assign individuals to their most probable cluster. Prior to analysis, we removed individuals where >20% of the data were missing. Clustering analyses were performed with *K* = 1–14 and repeated with 20 runs to assess the consistency of results. We used the admixture model with correlated allele frequencies; no *a priori* information on populations was incorporated in the analysis. Runs were performed using a burn-in period of 10^4^ followed by 10^5^ MCMC iterations. Results from the 20 runs were analyzed with CLUMPAK^43^. The estimated number of clusters (*K*) was taken to be that value of *K* with the highest probability of the data (Pr(X|*K*). This probability continuously increased from *K* = 1–12 (Fig. 6A), but it reaches a plateau and values of *K* higher than 7 increase the ‘assignation noise’; i.e. variance in individual assignations across repeated runs for a given *K*. At *K* = 7, the percentage of ‘mixed’ individuals, admixture reflecting shared ancestral polymorphism or recent introgression, is low (7%) (Fig. 6B), the differentiation between clusters is high (average F_ST_ = 0.25, p < 0.001), and the overall structure as divided among clusters remains stable across repeated runs. Based on these considerations, we chose this level of structuring as representative of isolated populations used in PIC analysis.

## Additional Information

**How to cite this article**: Greenfield, M. D. *et al*. Animal choruses emerge from receiver psychology. *Sci. Rep*. **6**, 34369; doi: 10.1038/srep34369 (2016).

## Supplementary Material

Supplementary Information

## Figures and Tables

**Figure 1:**
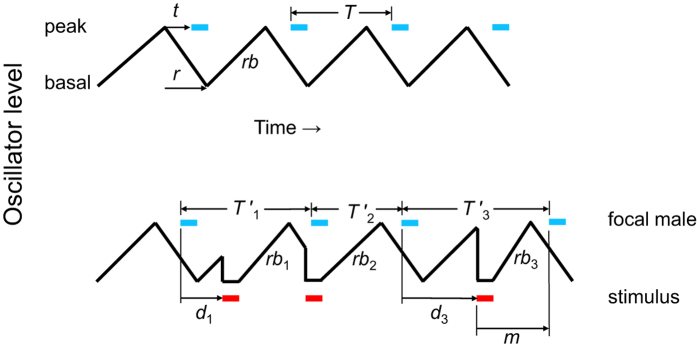
Inhibitory-resetting model for signal interactions between male neighbors in rhythmic acoustic species. Black sawtooth line in upper trace shows the periodic ascent (*rb*, rebound) and descent of the free-running central rhythm generator. After an effector delay *t* following ascent to the peak level, a call (thick blue dash) is broadcast; meanwhile the generator descends to its basal level over interval *r*. Calls are repeated rhythmically with a period *T*. Lower trace shows the same central rhythm generator as it is repeatedly inhibited and reset by a stimulus (male neighbor or acoustic playback; thick red dash). *T′* is the modified call period following a stimulus. The rebound *rb* from inhibition following a stimulus steepens when the stimulus occurs after a longer post-call delay *d*; *rb* is steepest (*rb*_3_) when the stimulus occurs just as the rhythm generator has ascended to its peak level (following post-call delay *d*_3_), yielding the shortest post-stimulus delay for the focal male’s next call. This minimum post-stimulus call delay is designated *m*. The model is adapted from ref. 21 and was derived from results in extensive playback experiments with various species, including *E. diurnus*.

**Figure 3:**
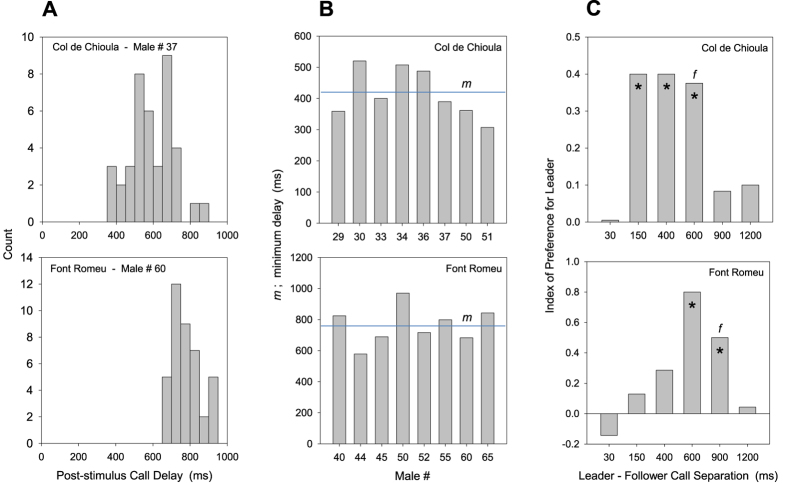
Timing parameters in male chorusing and female preference. **(A)** Call delay histograms of representative males from 2 populations (Col de Chioula, Font Romeu; Fig. 4A). The numbers of calls produced during 50-ms bins beginning at the onset of a synthetic call stimulus broadcast at random intervals are shown. The post-stimulus delay of the earliest of the male’s 40 calls following inhibition is equivalent to *m* in Fig. 1. (**B**) Minimum post-stimulus call delay, *m*, for each of 8 males tested via playback in the 2 populations. A male’s *m* is determined as the average of his 3 shortest delays. The population’s *m* is the average of the individual male values. **(C)** Index of preference by females in the 2 populations for the leading of 2 synthetic call stimuli broadcast in close succession. For a given population, the maximum leader-follower call separation at which the index remained ≥50% of the highest index value observed across all call separations was designated as *f*; in all but one population this highest index value was significant (H_o_: index = 0.0; 1-tailed sign test, α = 0.05; each female that preferred the leader in >50% of trials was labelled ‘+’ and labelled ‘−’ if < 50%; the sign test was performed on the labels of all females tested for responses to a given call separation). *Index of preference >0.0.

## References

[b1] Wilson E. O. Sociobiology: The New Synthesis (Harvard Univ. Press, Cambridge, MA, 1975).

[b2] Greenfield M. D. Mechanisms and evolution of communal sexual displays in arthropods and anurans. Adv Study Behav 35, 1–61 (2005).

[b3] Sismondo E. Synchronous, alternating, and phase‐locked stridulation by a tropical katydid. Science 249, 55–58 (1990). 17787627 10.1126/science.249.4964.55

[b4] Backwell P., Jennions M., Passmore N. & Christy J. Synchronized courtship in fiddler crabs. Nature 391, 31–32 (1998).

[b5] Moiseff A. & Copeland J. Firefly synchrony: a behavioral strategy to minimize visual clutter. Science 329, 181 (2010). 20616271 10.1126/science.1190421

[b6] Kahn A. T., Holman L. & Backwell P. R. Y. Female preferences for timing in a fiddler crab with synchronous courtship waving displays. Anim Behav 98, 35–39 (2014).

[b7] Walker T. J. Acoustic synchrony: two mechanisms in the snowy tree cricket. Science 166, 891–894 (1969). 17815755 10.1126/science.166.3907.891

[b8] Nityananda V. & Balakrishnan R. Synchrony during acoustic interactions in the bushcricket *Mecopoda* ‘chirper’ (Tettigoniidae: Orthoptera) is generated by a combination of chirp-by-chirp resetting and change in intrinsic chirp rate. J Comp Physiol A 193, 51–65 (2007). 10.1007/s00359-006-0170-1 16983544

[b9] Greenfield M. D. Cooperation and conflict in the evolution of signal interactions. Annu Rev Ecol Syst 25, 97–126 (1994).

[b10] Greenfield M. D. Signal interactions and interference in insect choruses: singing and listening in the social environment. J Comp Physiol A 201, 143–154 (2015). 10.1007/s00359-014-0938-7 25236356

[b11] Buck J. & Buck E. Mechanism of rhythmic synchronous flashing of fireflies. Science 159, 1319–1327 (1968). 5644256 10.1126/science.159.3821.1319

[b12] Hartbauer M., Haitzinger L., Kainz M. & Römer H. Competition and cooperation in a synchronous bushcricket chorus. Roy Soc Open Sci 1 **(2)**, 140167 (2014). 26064537 10.1098/rsos.140167 PMC4448899

[b13] Schwartz J. J. Male calling behavior, female discrimination and acoustic interference in the neotropical treefrog *Hyla microcephala* under realistic acoustic conditions. Behav Ecol Sociobiol 32, 401–414 (1993).

[b14] Murphy M. A., Thompson N. L. & Schul J. Keeping up with the neighbor: a novel mechanism of call synchrony in *Neoconocephalus ensiger* katydids. J Comp Physiol A 202, 225– 234 (2016). 10.1007/s00359-016-1068-1 26809565

[b15] Nityananda V. & Balakrishnan R. Modeling the role of competition and cooperation in the evolution of katydid acoustic synchrony. Behav Ecol 20, 484–489 (2009).

[b16] Miller C. T. & Bee M. A. Receiver psychology turns 20: is it time for a broader approach? Anim Behav 83, 331–343 (2012). 24013277 10.1016/j.anbehav.2011.11.025 PMC3763864

[b17] Greenfield M. D. & Schul J. Mechanisms and evolution of synchronous chorusing: emergent properties and adaptive functions in *Neoconocephalus* katydids (Orthoptera: Tettigoniidae). J Comp Psychol 122, 289–297 (2008). 18729657 10.1037/0735-7036.122.3.289

[b18] Greenfield M. D. & Roizen I. Katydid synchronous chorusing is an evolutionarily stable outcome of female choice. Nature 364, 618–620 (1993).

[b19] Marshall V. T. & Gerhardt H. C. A precedence effect underlies preferences for calls with leading pulses in the grey treefrog, Hyla versicolor. Anim Behav 80, 139–145 (2010). 20625471 10.1016/j.anbehav.2010.04.014 PMC2898283

[b20] Minckley R. L., Greenfield M. D. & Tourtellot M. K. Chorus structure in tarbush grasshoppers: inhibition, selective phonoresponse, and signal competition. Anim Behav 50, 579–594 (1995).

[b21] Greenfield M. D., Tourtellot M. K. & Snedden W. A. Precedence effects and the evolution of chorusing. Proc R Soc B Biol Sci 264, 1355–1361 (1997).

[b22] Spooner L. J. & Ritchie M. G. An unusual phylogeography in the bushcricket *Ephippiger ephippiger* from southern France. Heredity 97, 398–408 (2006). 16955113 10.1038/sj.hdy.6800884

[b23] Ritchie M. G. The shape of female mating preferences. Proc Nat Acad Sci USA 93, 14628–14631 (1996). 8962104 10.1073/pnas.93.25.14628 PMC26185

[b24] Barbosa F., Rebar D. & Greenfield M. D. Female preference functions drive inter-population divergence in male signaling: call diversity in the bushcricket *Ephippiger diurnus*. J Evol Biol 29, in press (2016). 10.1111/jeb.12940 27471011

[b25] Greenfield M. D., Siegfreid E. & Snedden W. A. Variation and repeatability of female choice in a chorusing katydid, *Ephippiger ephippiger*: an experimental exploration of the precedence effect. Ethology 110, 287–299 (2004).

[b26] Esquer-Garrigos Y., Greenfield M. D., Party V. & Streiff R. Characterization of 16 novel microsatellite loci for *Ephippiger diurnus* (Orthoptera: Tettigoniidae) using pyrosequencing technology and cross-species amplification. Eur J Entomol 113, 302– 306 (2016).

[b27] Felsenstein J. Phylogenies and the comparative method. Am Nat 125, 1–15 (1985). 10.1086/703055 31094602

[b28] Stone G. N., Nee S. & Felsenstein F. Controlling for non-independence in comparative analysis of patterns across populations within species. Phil Trans R Soc B Biol Sci 366, 1410–1424 (2011). 10.1098/rstb.2010.0311 PMC3081573 21444315

[b29] Anderson B., Ros P., Wiese T. J. & Ellis A. G. Intraspecific divergence and convergence of floral tube length in specialized pollination interactions. Proc R Soc B Biol Sci 281, art. no. 20141420 (2014). 10.1098/rspb.2014.1420 PMC4213615 25274360

[b30] Party V., Streiff R., Marin-Cudraz T. & Greenfield M. D. Group synchrony and alternation as an emergent property: elaborate chorus structure in a bushcricket is an incidental by-product of female preference for leading calls. Behav Ecol Sociobiol 69, 1957–1973 (2015).

[b31] Party V., Brunel-Pons O. & Greenfield M. D. Priority of precedence: receiver psychology, female preference for leading calls and sexual selection in insect choruses. Anim Behav 87, 175–185 (2014).

[b32] Reaney L. T., Sims R. A., Sims S. W. M., Jennions M. D. & Backwell P. R. Y. Experiments with robots explain synchronized courtship in fiddler crabs. Curr Biol 18, R62– R63 (2008). 18211839 10.1016/j.cub.2007.11.047

[b33] Camazine S., Deneubourg J.-L., Franks N. R., Sneyd J., Theraulaz G. & Bonabeau E. Self-organization in biological systems. (Princeton Univ. Press, Princeton, NJ, 2001).

[b34] Jang Y. & Greenfield M. D. Ultrasonic communication and sexual selection in wax moths: female choice based on energy and asynchrony of male signals. Anim Behav 51, 1095– 1106 (1996).

[b35] Hockham L. R., Graves J. A. & Ritchie M. G. Isolation and characterization of microsatellite loci in the bushcricket *Ephippiger ephippiger* (Orthoptera: Tettigoniidae). Mol Ecol 8, 905–906 (1999).

[b36] Hamill R. M., Noor M. A. F., Watson E. T. & Ritchie M. G. New microsatellite loci for the European bushcricket, Ephippiger ephippiger (Orthoptera: Tettigoniidae) Mol Ecol Notes 6, 340–342 (2006).

[b37] Schuelke M. An economic method for the fluorescent labeling of PCR fragments. Nature Biotech 18, 233–234 (2000). 10.1038/72708 10657137

[b38] Takezaki N., Nei M. & Tamura K. POPTREEW: Web version of POPTREE for constructing population trees from allele frequency data and computing some other quantities. Mol Biol Evol 31, 1622–1624 (2014). 24603277 10.1093/molbev/msu093

[b39] Midford P. E., Garland T. Jr. & Maddison W. P. *PDAP Package of Mesquite. Version 1.16* (2011).

[b40] Maddison W. P. & Maddison D. R. Mesquite: A Modular System for Evolutionary Analysis. *Version 3.04*. http://mesquiteproject.org (2015).

[b41] Garland T. Jr., Harvey P. H. & Ives A. R. Procedures for the analysis of comparative data using phylogenetically independent contrasts. Syst Biol 41, 18–32 (1992).

[b42] Pritchard J. K., Stephens M. & Donnelly P. Inference of population structure using multilocus genotype data. Genetics 155, 945–959 (2000). 10835412 10.1093/genetics/155.2.945 PMC1461096

[b43] Kopelman N. M., Mayzel J., Jakobsson M., Rosenberg N. A. & Mayrose I. Clumpak: a program for identifying clustering modes and packaging population structure inferences across K. Mol Ecol Res 15, 1179–1191 (2015). 10.1111/1755-0998.12387 PMC4534335 25684545

