# Supplementary material for: Animal choruses emerge from receiver psychology
Source: Sci Rep. 2016 Sep 27;6:34369. doi: 10.1038/srep34369 (PMC5037466; doi:10.1038/srep34369)
Supplement: Supplementary Information [file srep34369-s1.pdf]

Title: Animal choruses emerge from receiver psychology

Authors: Michael D. Greenfield[1*], Yareli Esquer-Garrigos[2,3], Réjane Streiff[2,3], and Virginie Party[1]

Supplementary note S1

Monte Carlo Simulation of Inhibitory Resetting

When 2 males sing with similar free-running rhythms and mutually adjust with the same resetting mechanism, simulation predicts calling in synchrony or alternation[20,21]. Synchrony occurs where singers rebound from inhibition with promptness similar to their rhythm during solo calling; alternation occurs where the rebound from inhibition is substantially faster. Via a chain reaction, the basic interaction between 2 neighboring males can yield synchrony that includes many individuals. By definition, though, out-of-phase alternation should be restricted to a pair of individuals. Paradoxically, the simple interaction initiated by an alternating pair spreads if individuals selectively attend to 1 or 2 neighbors only[20,44,45]. In these local groups a given singer alternates with his nearest neighbor(s) and – by default – synchronizes with his neighbor's neighbors (see Fig. 2), a feature that interlaces the small groups with each other and yields an expanding chorus.

The full model for the general inhibitory-resetting mechanism, as depicted in Fig. 1, is :

$$T' = s \cdot [(d + l/v) - (r - t)] + (T + \varepsilon) + (y - x), \text{ where :}$$

1) T' is the modified call period following interaction with a stimulus (or neighbor);

2) s is the duration of rhythm generator's rebound (rb_3 in Fig. 1) following inhibition at the latest possible time during the call cycle (after stimulus delay d_3 in Fig. 1) divided by the call period (T); it measures the greatest rapidity with which the generator can rebound from

inhibition relative to its rebound from basal level during free-running call cycles. *s* also represents the slope of the phase response curve (PRC), which regresses response phase [T' – T) / T] against stimulus phase (d / T) during interactive calling;

3) d is the delay between the focal male's concurrent call and the stimulus, measured as the interval from onset to onset;

4) l is the distance between the focal male and the stimulus;

5) v is the velocity of sound;

6) r is the interval during which the rhythm generator descends from its peak to its basal level during a free-running cycle;

7) t is the effector delay between the time when the rhythm generator attains its peak level and the onset of the triggered call;

8) T is the mean call period during a free running cycle;

9) ε is a stochastic element that accounts for intrinsic variation in the call period;

10) y is the length of the stimulus;

11) x is the length of the focal male's call.

References

20. Minckley, R.L., Greenfield, M.D. & Tourtellot, M.K. Chorus structure in tarbush grasshoppers: inhibition, selective phonoresponse, and signal competition. *Anim Behav* **50**, 579–594 (1995).

21. Greenfield, M.D., Tourtellot, M.K. & Snedden, W.A. Precedence effects and the evolution of chorusing. *Proc R Soc B Biol Sci* **264**, 1355–1361 (1997).

44. Greenfield, M. D. & Snedden, W. A. Selective attention and the spatio-temporal structure of orthopteran choruses. *Behaviour* **140**, 1–26 (2003).

45. Nityananda, V., Stradner, J., Balakrishnan, R. & Römer, H. Selective attention in a synchronising bushcricket: physiology, behavior and ecology. *J Comp Physiol A* **193**, 983–991 (2007).
